# Low‐carbohydrate diet score is associated with improved blood pressure and cardio‐metabolic risk factors among obese adults

**DOI:** 10.14814/phy2.15375

**Published:** 2022-07-13

**Authors:** Mohammad Sadegh Pour Abbasi, Niloofar Shojaei, Mahdieh Abbasalizad Farhangi

**Affiliations:** ^1^ Department of Cardiovascular Surgery Kashan University of Medical Sciences Kashan Iran; ^2^ School of Medicine Zanjan University of Medical Sciences Zanjan Iran; ^3^ Drug Applied Research Center Tabriz University of Medical Sciences Tabriz Iran

**Keywords:** cardio‐metabolic risk factors, low carbohydrate diet, metabolic syndrome, obesity

## Abstract

Obesity is associated with numerous co‐morbidities and diet, is one of the modifiable risk factors for prevention against these obesity‐related metabolic disorders. In the current study, we aimed to evaluate the association between adherence to low carbohydrate diet (LCD) score and serum lipids, glycemic markers, blood pressure, and anthropometric parameters among obese individuals. The current cross‐sectional study is a combination of two projects with total participants of 359 obese individuals (body mass index [BMI] ≥ 30 kg/m^2^) aged 20–50 years were included. Dietary intake was assessed by a validated semi‐quantitative food frequency questionnaire (FFQ) of 132 food items. Low carbohydrate diet score was estimated by deciles of dietary intakes. Metabolic syndrome (MetS) was defined based on the guidelines of the National Cholesterol Education Program Adult Treatment Panel III (NCEP‐ATP III). Enzymatic methods were used to assess serum lipids, glucose, and insulin concentrations. Blood pressure was measured by sphygmomanometer and body composition with bioelectrical impedance analysis (BIA). Higher adherence to LCD score was associated with significantly lower DBP and triglyceride (TG) concentrations and increased high density lipoprotein (HDL)‐C levels after adjustment for the confounders (*p* < 0.05). A non‐significant reduction in systolic blood pressure (SBP) and total cholesterol (TC) values were also observed. Also, high adherence to LCD score was associated with reduced prevalence of metabolic syndrome (*p* < 0.05). Higher BMI, fat mass, and lower fat‐free mass were also accompanied with higher adherence to LCD score. According to our study, low carbohydrate diet score was associated with more favorable cardio‐metabolic risk factors independent of some confounders like age, BMI, sex, and physical activity level. Further studies in different communities will help for generalization of our findings.

## 1 INTRODUCTION

The prevalence of non‐communicable disease (NCDs) is increasing worldwide due to changes in lifestyle and dietary intakes; alongside with increased prevalence of NCDs, obesity is considered as one of the most important risk factors of metabolic disorders. The worldwide number of overweight and obese adults in 2014, was more than 1.9 billion and 600 million adults respectively (WHO, 2015). Similarly, in Iran, increased obesity prevalence mostly due to nutrition transition has been occurred and the combined prevalence of overweight and obesity may be as high as 76% in some regions (Jafari‐Adli et al., 2014). Diet, is a modifiable risk factor of chronic disease and in recent years, numerous studies have been published focusing on the role of dietary indices or dietary patterns as an alternative approach to single nutrient evaluation in investigating the diet‐disease relationships (Lagiou et al., 2007; Mirmiran et al., 2010; Sangsefidi, Salehi‐Abarghouei, et al., 2021; Sjogren et al., 2010; Trichopoulou et al., 2007). Dietary carbohydrate is the main source of usual daily energy intake in Asian countries and numerous studies in China (Dehghan et al., 2017; Villegas et al., 2007), Korea (Ha, Kim, et al., 2018; Kim et al., 2008; Song et al., 2014) and Iran (Hosseinpour‐Niazi et al., 2013; Mirmiran et al., 2016) had found the role of high amount of dietary carbohydrate intake and increased risk of metabolic disorders and cardiovascular events among different populations. Therefore, different recommendations of dietary carbohydrate restriction for weight loss in different communities are available (Browning et al., 2011; NHLBI Obesity Education Initiative Expert Panel on the Identification, Evaluation, and Treatment of Obesity in Adults (US), 1998; Ruaño et al., 2006). However, there are several inconsistencies about the association between reduced dietary carbohydrates and metabolic disease and it seems that these studies should be performed in different regions; for example, in the study by Seidelmann SB et al. (Seidelmann et al., 2018), among 15,428 American adults, both low carbohydrate consumption (<40%) and high carbohydrate consumption (>70%) were associated with greater mortality risk in a U shaped manner. In another study, the author suggested that dietary restriction of only some types of carbohydrate is beneficial for cardiovascular health (Volek et al., 2008). And in other study in Mexico, moderate carbohydrate intake had been introduced as a factor of improving dyslipidemia because of high fiber intake (Jiménez‐Cruz et al., 2004). In one study in China, there was a different effect of white rice consumption on metabolic markers of diabetes and dyslipidemia in different regions (Dong et al., 2015). The concept of low carbohydrate diet score (LCD) first identified by Halton TL et al. (Halton et al., 2008), and then has been used and modified in several studies. LCD is a relatively new concept that is a suggested approach of macronutrient diet scores and provides a comprehensive approach of diet–disease associations and is more suitable to explain the relation between diet and risk of chronic diseases (de Koning et al., 2011; Halton, Willett, Liu, Manson, Albert, Rexrode, & Hu, 2006). LCD considers the proportion of targeted dietary macronutrients in the form of a dietary pattern and is defined as a diet with lower intakes of carbohydrates and higher intakes of fats (Hite et al., 2011; Jafari‐Maram et al., 2019). Several studies have revealed the association between low carbohydrate diet score with metabolic disorders like type 2 diabetes (Halton et al., 2008), coronary heart disease (Halton, Willett, Liu, Manson, Albert, Rexrode, et al., 2006), some types of cancer (Nilsson et al., 2013; Song et al., 2018), and metabolic syndrome (Ha, Joung, & Song, 2018). But, just a very limited number of studies have focused on low carbohydrate diet score and its effects on cardiovascular biomarkers among obese individuals. Moreover, almost no study is available to evaluate this hypothesis in Tabriz city of Iran. Therefore, in the current study, we aimed to investigate the adherence to LCD diet score and its association with lipid profile, glycemic markers, blood pressure, and metabolic syndrome among obese adults that were recruited from two previously performed projects among obese individuals of Tabriz, Iran (Abbasalizad Farhangi et al., 2020; Farhangi et al., 2020; Khodarahmi et al., 2019).

## 2 METHODS AND MATERIALS

### 2.1 Participants

**FIGURE 1:**
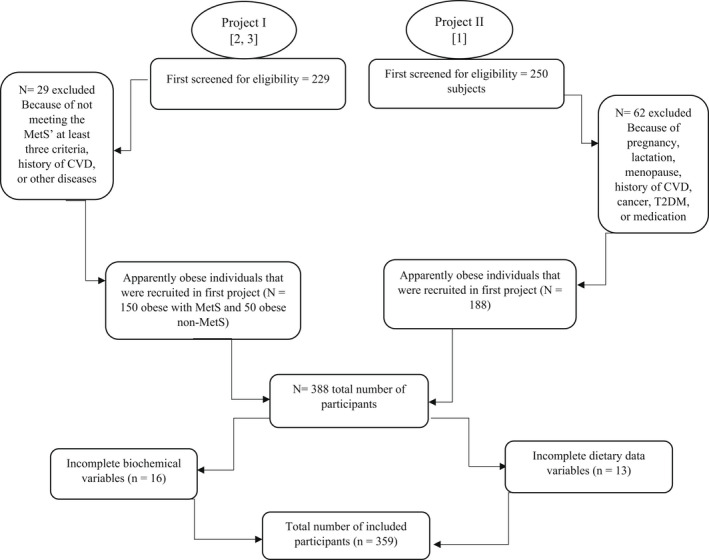
Study flow chart of participants' recruitment.

The current cross‐sectional study is a combination of two previously recruited projects in Iran that was conducted among 359 obese individuals (57.9% males and 41.5% females) in Tabriz, Iran. The participants were recruited from the combination of two projects among obese individuals (Abbasalizad Farhangi et al., 2020; Farhangi et al., 2020; Khodarahmi et al., 2019). Figure 1, presents the study flowchart. Study subjects were invited by public announcements and were included if they met inclusion criteria (being aged 20–50 years old, BMI ≥ 30 kg/m^2^). The age range was selected because of the effects of both aging and menopause on the cardio‐metabolic risk factors; serum lipids and fasting blood sugar are increased in older adults and in postmenopausal women (Frishman et al., 1992; Kawanishi et al., 2018; Stevenson et al., 1993). Also, according to WHO definition, 20–60 years is defined as adult ages (World Health Organization (WHO), 2020); since menopause occurs usually at age 50, therefore we removed those aged over 50 from women and also men to remove the confounding effects of age in both genders. While the exclusion criteria were: Being pregnant, lactating, menopause, having recent bariatric surgery, or cardiovascular disease (CVD), cancer, hepatic and renal diseases, diabetes mellitus, and taking any weight affecting medications. Full‐informed approved written informed consent was taken from all of the participants and the study proposal was approved by the Ethics Committee of Tabriz University of Medical Sciences, Tabriz, Iran (Registration number: IR.TBZME‐D.REC.1400.454). IR.TBZMED.REC.1396.768.

### 2.2 General characteristics and anthropometric assessments

Socio‐demographic information including sex, age, smoking status, education attainment, marital status, occupation, medical histories, and family size were obtained via questionnaire; then, socioeconomic status (SES) score was calculated. Body composition measurements were done by bioelectrical impedance analysis (BIA) method (Tanita, BC‐418 MA, Tokyo, Japan). Participant's height and weight were measured using a wall‐mounted stadiometer and a Seca scale (Seca co., Hamburg, Germany) to the nearest 0.5 cm and 0.1 kg respectively. Short form of the International Physical Activity Questionnaire (IPAQ) was used for physical activity assessment (Vasheghani‐Farahani et al., 2011). Waist circumference (WC) was measured at the midpoint between the lower costal margin and the iliac crest using a tape measure to the nearest 0.1 cm while hip circumference (HC) was measured over the widest part of the buttocks and was recorded to the nearest 0.1 cm. BMI and waist‐to‐hip ratio (WHR) were calculated. Blood pressure was measured with a standard mercury sphygmo‐manometer twice in the same arm after at least 15 min of rest and then mean of the two measurements was used for analysis. Metabolic syndrome (MetS) was defined according to the NCEP‐ATP III criteria (Grundy et al., 2005).

### 2.3 Dietary assessments

Dietary information was collected using a validated semi‐quantitative food frequency questionnaire (FFQ), adapted for Iranian population (Mirmiran et al., 2010). Participants were asked to report frequency and amount of each food item consumed on a daily, weekly, monthly or yearly basis. Then, the reported frequency of consumed foods and portion sizes for each food items were converted to grams using household measures. Daily energy and nutrient contents were analyzed using the US Department of Agriculture's (USDA) national nutrient databank. Nevertheless, some local food items that were not available in USDA's food composition table (FCT), was analyzed using The Iranian FCT. All of the dietary variables were energy‐adjusted by residual method (Willett & Stampfer, 1986). For LCD score, energy‐adjusted carbohydrate, fat, and protein intakes were categorized into deciles. Then, descending energy‐adjusted deciles of carbohydrate intake and ascending deciles of energy‐adjusted fat intakes were labeled into 1–10 and were summed to achieve final LCD score ranged from 2 to 20 points, this method is used in several previous studies (Lagiou et al., 2007; Sjogren et al., 2010; Trichopoulou et al., 2007).

### 2.4 Biochemical assessment

10 ml venous blood samples were obtained from each subject and blood samples were centrifuged at 4500 rpm for 10 min to separate serum and plasma samples. Serum total cholesterol (TC), triglyceride (TG), high‐density lipoprotein cholesterol (HDL‐C), and fasting blood sugar (FBS) were evaluated using a commercial kit (Pars Azmoon). Furthermore, low‐density lipoprotein cholesterol (LDL‐C) level was estimated by the Friedewald Equation (Halton, Willett, Liu, Manson, Albert, Rexrode, & Hu, 2006). Enzyme‐linked immunosorbent assay kits were used to measure serum insulin, concentrations (Bioassay Technology Laboratory, Shanghai Korean Biotech). Homeostatic model assessment for insulin resistance (HOMA‐IR) was calculated using the formula: fasting insulin (μ IU/ml) × fasting glucose (mmol/L) /22.5 and quantitative insulin sensitivity check index QUICKI as 1/[log fasting insulin (μU/ml) + log glucose (mmol/L)].

### 2.5 Statistical analyses

Statistical analysis of the data was performed using Statistical Package for Social Sciences (version 21.0; SPSS Inc.,) at a statistical significance level of <0.05. Data are presented as frequency (%) for categorical variables and mean ± standard deviation (SD) for continuous variables. The differences in discrete and continuous variables across different quartiles of LCD scores were compared using Chi‐square test and one‐way ANOVA respectively. ANCOVA was used for comparison of biochemical variables after adjustment for confounders (age, gender, BMI, PA).

## 3 RESULTS

**FIGURE 2:**
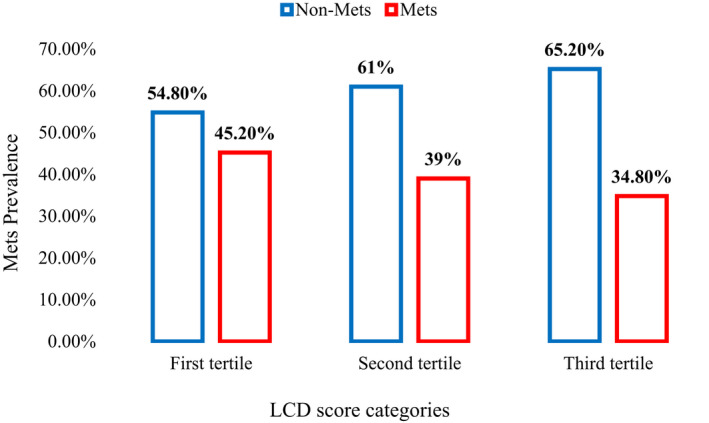
The prevalence of metabolic syndrome in different LCD score categories (*p* < 0.05 by chi‐square analysis).

The comparison of energy and macronutrient intakes among different LCD score categories is presented in Table 1. As expected, there is a significant difference in ascending order of fat intake and descending order of carbohydrate intake among LCD score categories (*p* < 0.001). Table 2 presents the comparison of demographic and anthropometric variables among different LCD score categories. As presented, those with the highest adherence to LCD score were younger (*p* < 0.001), had higher BMI (*p* = 0.003), lower FFM (*p* = 0.022) and lower BMR (*p* = 0.023) compared with those with the lowest adherence to LCD score. Moreover, Table 3 shows that higher adherence to LCD score, significantly reduces DBP values and TG concentrations while increasing HDL levels (*p* < 0.05). Although, SBP and TC values were also decreased with higher adherence to LCD, but, this reduction was not statistically significant. No significant difference in insulin, HOMA‐IR, and QUICKI values were observed across LCD tertiles (*p* > 0.05). The prevalence of metabolic syndrome across different tertiles of LCD score (Figure 2) presents that high adherence to LCD diet score is associated with reduced prevalence of metabolic syndrome (*p* < 0.05).

**TABLE 1:** The comparison of energy and macronutrient intakes of study population by dietary LCD tertiles

Variable	*N*	Mean	SD	*p*‐value
Energy (kcal)				
1st	125	2892.88	966.45	0.191
2nd	102	3005.23	1167.99	
3rd	132	3141.18	1154.73	
Protein (%)				
1st	125	13.26	2.06	0.143
2nd	102	13.31	1.79	
3rd	132	12.72	1.98	
CHO (%)				
1st	125	67.51	4.20	<0.001
2nd	102	60.35	1.96	
3rd	132	52.90	4.19	
Fat (%)				
1st	125	23.08	2.68	<0.001
2nd	102	28.66	1.67	
3rd	132	36.36	4.82	

*Note*: All of the macronutrients are reported as percentage of total calorie intake.

Abbreviations: CHO, carbohydrate.

**TABLE 2:** General characteristics of study population by dietary LCD tertiles

Variable	*N*	Mean	SD	*p* value
Age (y)				
1st	125	43.44	9.02	<0.001
2nd	102	39.19	8.97	
3rd	132	38.91	8.91	
Sex (male)				
1st	125	43.44	9.02	0.335
2nd	102	39.19	8.97	
3rd	132	38.91	8.91	
SES score				
1st	125	10.56	2.37	0.104
2nd	102	9.56	2.76	
3rd	132	9.80	2.42	
BMI (kg/m^2^)				
1st	125	31.61	4.77	0.003
2nd	102	32.65	4.96	
3rd	132	33.66	4.60	
WC (cm)				
1st	125	105.78	9.69	0.423
2nd	102	107.03	9.24	
3rd	132	107.28	9.71	
WHR				
1st	125	0.93	0.06	0.586
2nd	102	0.94	0.07	
3rd	132	0.92	0.08	
FM (%)				
1st	125	32.43	7.62	0.434
2nd	102	34.32	9.97	
3rd	132	34.36	9.52	
FFM (%)				
1st	125	66.20	12.34	0.022
2nd	102	60.50	10.81	
3rd	132	60.80	12.66	
BMR (kcal)				
1st	125	8361.58	1454.24	0.023
2nd	102	7556.91	1716.71	
3rd	132	7706.85	1609.93	
PA (MET‐min/week)				
1st	125	2063.92	3532.49	0.960
2nd	102	2247.38	3485.71	
3rd	132	2177.49	2919.13	

*Note*: All data are mean (±SD) or percentage. *p* values derived from one‐Way ANOVA with Tukey's *post‐hoc* comparisons.

Abbreviations: BMR, basal metabolic rate; PA, physical activity; SES, socio‐economic status; WC, waist circumference; WHR, waist to hip ratio; FM, fat mass.

**TABLE 3:** Biochemical variables of study population by dietary LCD tertiles

Variables	*N*	Mean	SD	*p* value
SBP (mm Hg)				
1st	125	124.84	14.52	0.078
2nd	102	123.39	15.24	
3rd	132	120.35	17.97	
DBP (mm Hg)				
1st	125	83.41	9.79	**0.039**
2nd	102	82.08	11.10	
3rd	132	79.73	13.46	
FBS (mg/dl)				
1st	125	92.40	16.03	0.904
2nd	102	93.59	28.45	
3rd	132	92.61	14.96	
TC (mg/dl)				
1st	125	194.97	38.26	0.377
2nd	102	187.80	37.77	
3rd	132	190.91	34.78	
TG (mg/dl)				
1st	125	163.72	96.02	**0.046**
2nd	102	154.71	81.37	
3rd	132	135.37	95.92	
HDL (mg/dl)				
1st	125	43.11	9.53	**0.025**
2nd	102	41.60	8.83	
3rd	132	45.13	9.68	
LDL (mg/dl)				
1st	125	126.75	32.88	0.347
2nd	102	122.44	29.58	
3rd	132	121.08	32.60	
Insulin (μIU/L)				
1st	125	17.22	2.34	0.67
2nd	102	15.34	4.65	
3rd	132	15.75	5.12	
HOMA‐IR				
1st	125	3.96	0.43	0.77
2nd	102	3.73	0.78	
3rd	132	3.63	1.09	
QUICKI				
1st	125	0.33	0.044	0.21
2nd	102	0.32	0.027	
3rd	132	0.32	0.029	

*Note*: All data are mean (±SD). *p* values derived from ANCOVA after adjustment for confounders (age, gender, BMI, PA).

Abbreviations: DBP, diastolic blood pressure; HDL‐C, high density lipoprotein cholesterol; HOMA‐IR, homeostatic model assessment for insulin resistance; LDL‐C, low density lipoprotein cholesterol; QUICKI, quantitative insulin sensitivity check index; SBP, systolic blood pressure; TC, total cholesterol; TG, triglyceride.

## 4 DISCUSSION

In the current cross‐sectional study among 359 obese adults, high adherence to LCD score was associated with more favorable CVD risk factors including higher HDL and lower DBP and TG values. Also, the prevalence of MetS reduced by increased adherence to LCD score. Similar to our study, in the study by Sangesefidi ZS et al. (Sangsefidi, Lorzadeh, et al., 2021), LCD score was associated with lower odds of metabolic syndrome and higher HDL concentrations. Also, Ha K et al. (Ha, Joung, & Song, 2018), reported that LCD diet did not increase the risk of metabolic syndrome among Korean adults and reduced the risk of low HDL‐cholesterol values. These findings will be explained one by one. Dietary carbohydrate is a key regulator of fatty acid metabolism; dietary carbohydrate stimulates insulin secretion and de novo lipogenesis; in dietary carbohydrate restriction, even in the presence of high saturated fatty acid intake (such as LCD diet), insulin secretion and glucose, fructose and insulin ligands availability will be reduced; consequently, increased fat oxidation, decreased lipogenesis, and decreased secretion of very low‐density lipoprotein (VLDL)‐C will occur (Volek et al., 2008). Reduced TG and increased HDL as observed in our study, is explained by this mechanism.

In our study, LCD score was associated with significant reduction in DBP and a clinically significant but statistically non‐significant reduction in SBP. Low carbohydrate diet is capable to improve arterial function and enhanced endothelial nitric oxide synthase (eNOS) phosphorylation; in the study by Bosse JD et al. (Bosse et al., 2013), a low‐carbohydrate/high‐fat diet reduced blood pressure and improved arterial function in six‐week‐old spontaneously hypertensive. Improved endothelium‐dependent (acetylcholine) relaxation of mesenteric arteries, reduced contraction (potassium chloride, phenylephrine), and increased phosphorylation of eNOS^Ser1177^ in arteries were reported as mechanisms of reduced blood pressure after LCD diet in their study. They also reported reduced plasma glucose, insulin, and HOMA‐IR, although we did not find any statistically significant difference in serum glucose or insulin level of highest versus lowest LCD categories, but, we found a significantly lower prevalence of MetS in those with highest adherence to LCD score. Similarly, numerous studies reported lower chance of MetS with higher adherence to low carbohydrate diet or with dietary carbohydrate restriction (Ha, Joung, & Song, 2018; Hyde et al., 2019; Kim et al., 2008; Sangsefidi, Lorzadeh, et al., 2021; Volek et al., 2008). In a nationwide cohort study by Li Q et al. (Li et al., 2021), there was a *U*‐shaped association between the percentage of carbohydrate intake (mean, 56.7%; SD, 10.7) and new‐onset hypertension (*p* < 0.001), with the lowest risk observed at 50%–55% carbohydrate intake.

In our study, high adherence to LCD score was associated with higher BMI, higher fat mass, and lower fat‐free mass; this might be as unfavorable result because of higher prevalence of obesity; this is not surprising because high fat intake is associated with higher obesity prevalence, similar to our finding, in the study by Nilsson LM et al. (Nilsson et al., 2013), low carbohydrate diet score was associated with higher BMI among 62,582 individuals (*p* < 0.001). This finding was also similar to the study by Na HY et al. (Na et al., 2012) that reported higher BMI in those with higher adherence to LCD scores among 20,717 Chinese subjects aged 45–59 years. LCD scoring in the current study was not a weight loss regimen as approved by previous studies; in other words, weight loss and reduced BMI should be accompanied by low and very low percentage energy from carbohydrate (≥10% to less than 50% of energy) (Trichopoulou et al., 2007), whereas, in our study population, consumption of carbohydrates, even at the lowest category of LCD score distribution (52.90%), was higher than that advocated by the prescribed low‐carbohydrate diets for weight management.

In conclusion, our cross‐sectional study demonstrated favorable effects of low carbohydrate diet scores in modifying serum lipids and blood pressure among a representative sample of obese adults. Furthermore, community‐based studies in different geographical distributions are necessary to generalize these findings.

## AUTHOR CONTRIBUTIONS

All authors approved the final version of the article. Mahdieh Abbasalizad Farhangi designed the study and served as a supervisor for this research. Mahdieh Abbasalizad Farhangi wrote and revised the manuscript. Mahdieh Abbasalizad Farhangi also performedthe statistical analysis.

## FUNDING INFORMATION

The work has been granted by Research Undersecretary of Tabriz University of Medical Sciences.

## CONFLICT OF INTEREST

Authors declare that there is no conflict of interest.

## ETHICS STATEMENT

All subjects provided a written informed consent before participation in the study. The study protocol was approved and registered by the ethics committee of Tabriz University of Medical Sciences (Registration number: IR.TBZMED.REC.1396.768). We confirm that methods were performed in accordance with the declaration of Helsinki's guidelines and regulations.
